# Viral and non-viral cellular therapies for neurodegeneration

**DOI:** 10.3389/fmed.2025.1718669

**Published:** 2026-01-09

**Authors:** Jyotsna Srivastav, Sachin Sharma

**Affiliations:** Department of Biosciences, Graphic Era University, Dehradun, India

**Keywords:** neurodegeneration, gene therapy, stem cell therapy, antisense oligonucleotides (ASOs), adeno-associated virus (AAV)

## Abstract

Neurodegenerative diseases such as Alzheimer's disease (AD), Parkinson's disease (PD), Huntington's disease (HD), and amyotrophic lateral sclerosis (ALS) are characterized by progressive loss of neurons and still lack curative treatment options. In this review, we describe current and developing therapeutic strategies that include viral vector-based gene delivery, antisense oligonucleotide (ASO) and RNA interference methods, stem cell transplantation, and genome editing technologies. Adeno-associated viruses (AAVs) and lentiviruses have been used for gene delivery in preclinical and clinical studies, while ASOs are under development to reduce expression of pathogenic proteins such as tau, α-synuclein, and mutant huntingtin. Cellular therapies, including mesenchymal stem cell (MSC)-based paracrine support and transplantation of neurons derived from induced pluripotent stem cells (iPSCs), are being evaluated, particularly in PD and AD. We also discuss important gene targets such as APOE4, GBA1, SCNA, and MAPT, and how treatment strategies may differ between monogenic and polygenic forms of these disorders. Lastly, we highlight recent efforts focused on genes like TREM2, PINK1, and progranulin, and examine their role in the future development of gene- and cell-based interventions.

## Graphical Abstract

**Figure:**
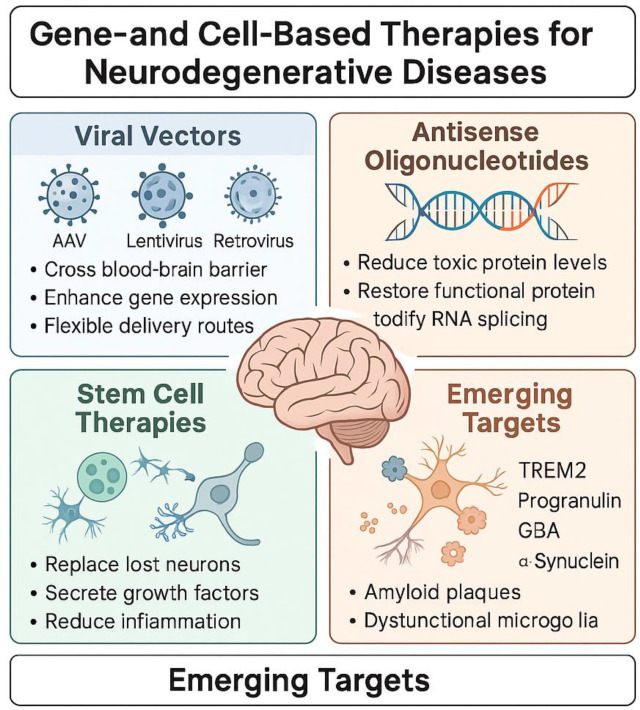

## Introduction

Neurodegenerative diseases—including Alzheimer's disease (AD), Parkinson's disease (PD), Huntington's disease (HD), and amyotrophic lateral sclerosis (ALS)—are characterized by progressive neuronal loss in the central and peripheral nervous systems. This degeneration leads to a range of functional impairments, including cognitive decline, memory deficits, bradykinesia, motor dysfunction, and peripheral neuropathy (1, 2). Although therapeutic research has advanced over recent decades, most available treatments remain symptomatic, without modifying the underlying disease course.

**Figure 1:**
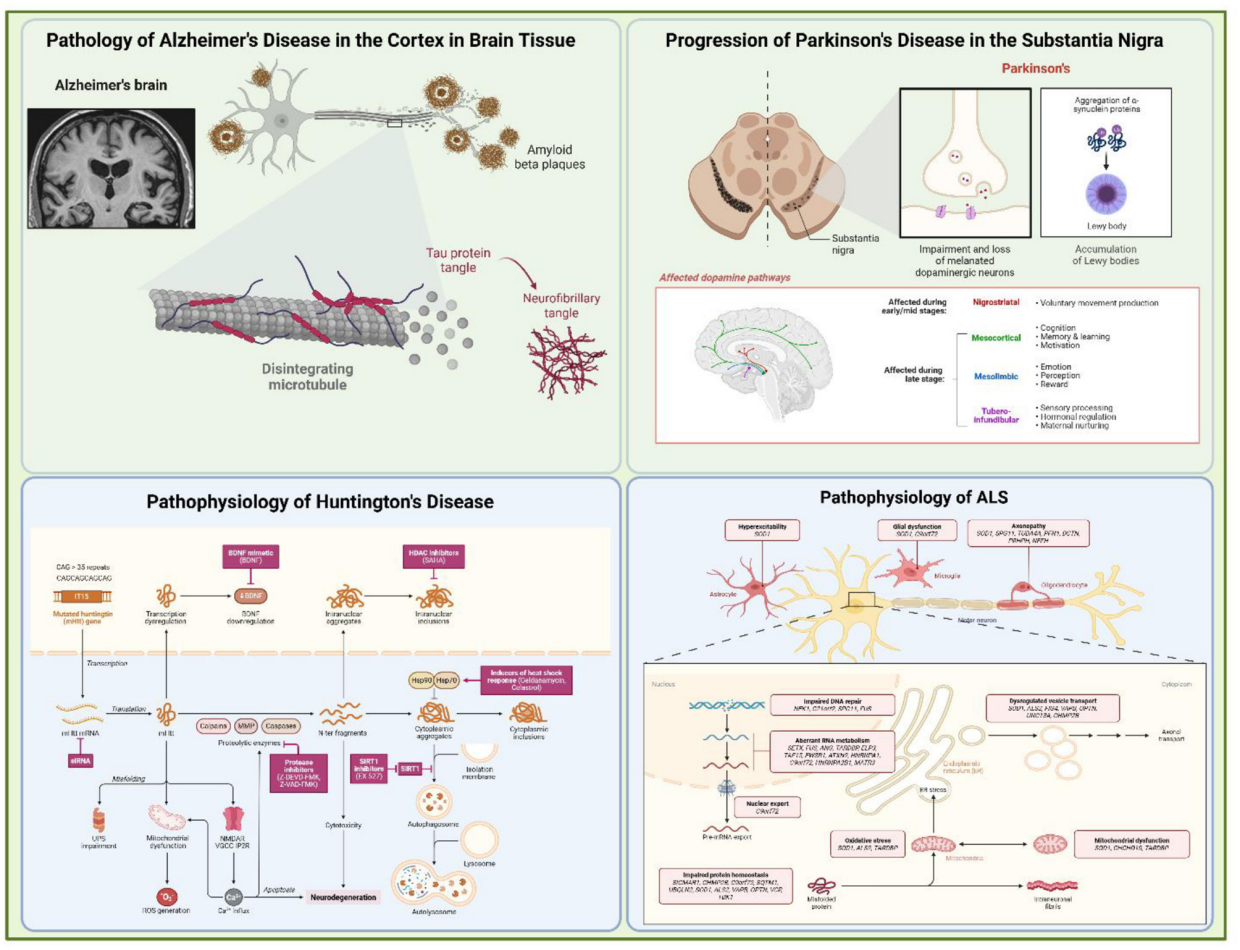
A schematic diagram of major neurodegenerative disorders and their mechanisms in pathophysiology. The diagram shows Alzheimer's, Parkinson's, Huntington's, and Amyotrophic lateral sclerosis disorders. The common symptoms among them lie cognitive dysfunction, memory deficits, and direction loss. These disorders are monogenic and polygenic in several cases.

AD is pathologically defined by extracellular β-amyloid (Aβ) deposition and intracellular accumulation of hyperphosphorylated tau protein, particularly in cortical and hippocampal regions. In contrast, PD is marked by dopaminergic neuronal loss in the substantia nigra and basal ganglia, resulting in both motor and non-motor symptoms. While AD and PD account for most neurodegenerative diagnoses globally, HD and ALS—though less common—present distinct pathophysiological mechanisms that are now being investigated using gene and cell-based approaches (3, 4) (Figure 1).

Due to the limited success of conventional pharmacological treatments, several experimental strategies are under development. These include gene replacement or suppression using viral vectors, gene expression modulation through antisense oligonucleotides (ASOs), and stem cell-based approaches aimed at neuronal replacement or paracrine support (5, 6). Among viral vectors, adeno-associated viruses (AAVs) and lentiviruses have shown encouraging results for delivering therapeutic genes across the blood-brain barrier in both preclinical models and early clinical studies (7–9). For example, AAV9 exhibits central nervous system tropism and has been used in disorders such as spinal muscular atrophy and PD (10, 11). Similarly, ASOs are under investigation for silencing pathogenic targets such as mutant huntingtin in HD and tau in AD (12, 13).

Stem cell therapies, including those based on mesenchymal stem cells (MSCs), neural stem cells (NSCs), and induced pluripotent stem cells (iPSCs), are being explored for both cell replacement and secretion of neuroprotective or immunomodulatory factors. Early-phase clinical trials have demonstrated the feasibility of transplanting iPSC-derived dopaminergic neurons in PD, while MSCs are being studied for their ability to modulate neuroinflammation in AD and ALS models (14, 15).

**Figure 2:**
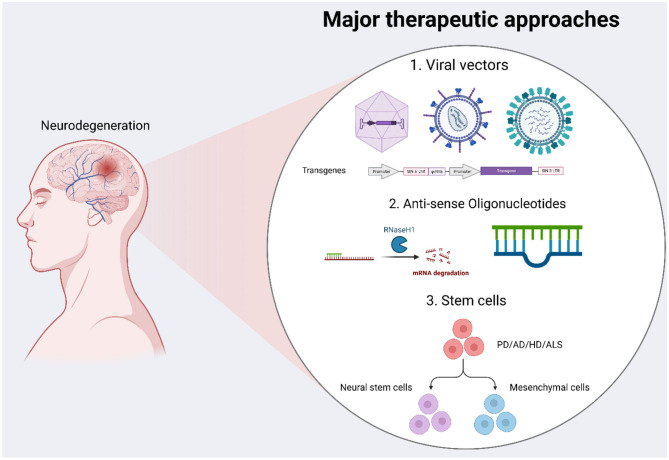
It shows major therapeutic approaches that are underway for the treatment of neurodegeneration. We mentioned viral vectors carrying different transgenes, short oligonucleotides, and differentiated stem cells expressing dopaminergic neurons or replacing neurofibrillary tangles in Alzheimer's disease.

This review outlines therapeutic strategies under investigation, including viral vector delivery, RNA-based methods, stem cell transplantation, and genome editing tools. We focus particularly on AD and PD, while also covering HD, ALS, and MS where relevant (Figure 2). In addition, we examine emerging genetic targets, discuss therapeutic considerations for monogenic vs. polygenic disorders, and explore how combined or sequential therapies may contribute to future developments in neuro-regenerative treatment.

## Viral vectors for gene-transferring tools

The onset and progression of neurodegenerative diseases such as Alzheimer's disease (AD) and Parkinson's disease (PD) are influenced by genetic mutations and environmental or epigenetic factors that affect gene expression at both local and systemic levels. With advancements in vector design and molecular medicine, viral vector-based gene therapy has become an important approach for delivering therapeutic genes to the central nervous system (CNS). This section introduces the main viral platforms—adeno-associated virus (AAV), lentivirus, and gamma-retrovirus—that have shown relevance for neurodegenerative disease treatment (Table 1).

**Table 1:** A table summarizing active clinical trials for neurodegenerative disorders.

**Sr. no**.	**Disease**	**Drug name**	**Viral vector**	**Therapeutic gene**	**Administration method**	**Phase**	**Status**
1.	Alzheimer's disease	AAV2-BDNF	AAV2	BDNF	Stereotactic injection	1	Recruiting
		LX1001	AAVrh10	APOE2	Intrathecal	1, 2	Recruiting
2.	Parkinson's disease	AAV-GAD	AAV2	GAD	Subthalamic infusion	1, 2	Recruiting
		AAV2-GDNF	AAV2	GDNF	Image-guided infusion	1	Active, not recruiting
		VY-AADC02	AAV2	AADC	ND	2	Active, not recruiting
		LY3884961	AAV9	GBA1	Intra-cisterna magna	1, 2	Recruiting
3.	Huntington's disease	AB-1001	AAVrh10	CYP46A1	Intracerebral injection	1, 2	Active, not recruiting
		AMT-130, rAAV5-miHTT	AAV5	HTT	Stereotactic infusion	1, 2	Recruiting

### Adeno-associated virus

Adeno-associated virus (AAV) vectors have been widely used for CNS gene delivery due to their tissue-specific tropism, relatively low immunogenicity, and ability to maintain transgene expression over time (16). The efficiency of AAVs depends on their capsid composition and the design of the inserted transgene (17). Advances in capsid engineering—using approaches such as directed evolution and rational design—have improved ability of AAVs to transduce target tissues (18). For production, AAVs are typically manufactured in HEK-293 or HeLa cells, or by using replication-deficient herpes simplex virus (HSV) systems (19, 20).

A major limitation in AAV-based therapy for AD and PD is the difficulty in crossing the blood-brain barrier (BBB). Many vectors that perform well *in vitro* fail to reach therapeutic levels in the brain *in vivo*. To address this, direct delivery routes—such as intraparenchymal, intracerebral, and intravenous administration—have been tested using AAVs with enhanced BBB permeability. Among them, AAV9 has been studied extensively for its ability to reach CNS tissue via vesicle-mediated transport (21). Newer optimized variants like AAV-DJ, AAV-CAP-B22, AAV-MaCPNS1, and humanized AAV9-PhP.B have demonstrated improved CNS and peripheral nervous system (PNS) transduction (22–25).

AAVs based cellular therapies have entered in clinical applications for several neurodegenerative diseases such as Alzheimer's, Parkinson's, and Batten disorders (Table 1). The accumulation of soluble Aβ oligomers (AβO) in brain cortex regions causes synapse failure and memory impairment in Alzheimer's disease. The introduction of AAV-NUsc1, a single-chain variable-fragment antibody (scFv) that selectively inhibited binding of AβO binding to neurons, protecting synapses and rescuing memory in Alzheimer's mice (26).

Batten disease, a rare and inherited disorder, is a collection of several forms of genetic conditions, and CLN2 is a specific genetic mutation subtype. AAVrh.10 and AAV9 vectors have been used to deliver CLN2 and CLN6 transgenes in clinical trials (27, 28). In PD, AAV2 has been used to deliver genes like aromatic L-amino acid decarboxylase (AADC) and glial cell line-derived neurotrophic factor (GDNF), which have shown functional benefits (29, 30). In spinal muscular atrophy, AAV9 is employed to deliver the SMN gene, which supports motor neuron survival (31).

### Lentivirus

Lentiviral vectors, derived from HIV-1, are widely used for gene therapy because they can integrate into the host genome and transduce both dividing and non-dividing cells. Modern lentiviral systems often use third-generation, self-inactivating backbones to reduce safety risks while maintaining efficacy (32). These vectors support relatively large transgenes (~9 kb) and have been optimized for stability and immune evasion.

To enhance safety and transduction efficiency, lentiviruses are engineered with the VSV-G envelope protein and lack accessory genes such as vif, vpu, vpr, and nef (33–35). They are particularly useful for targeting hematopoietic and neural cells. One of the early studies involved delivery of the Bcl-xL gene, which helped prevent apoptosis in cholinergic neurons (36). In Parkinson's disease (PD), lentiviral mediated delivery of dopamine synthesis enzymes via ProSavin platform showed clinical safety and motor improvement in the advance cases (37).

## Nucleic acid-based drugs

In contrast to viral vectors, nucleic acid-based drugs provide a therapeutic alternative that avoids risks such as insertional mutagenesis and immune responses related to viral components. These synthetic molecules act on RNA transcripts and have gained attention following recent clinical approvals and encouraging results in preclinical studies (38).

Antisense oligonucleotides (ASOs) are short, chemically modified nucleotides that bind to complementary RNA sequences to regulate splicing, affect transcript stability, or inhibit translation. ASOs have been studied in several neurodegenerative diseases, including tau-directed ASOs in Alzheimer's disease and those targeting mutant huntingtin in Huntington's disease (13, 39, 40). Mechanistically, ASOs are categorized into two groups: protein-lowering ASOs, which reduce expression of disease-related proteins like tau or huntingtin, and protein-restoring ASOs, which increase production of functional proteins such as survival motor neuron (SMN) in spinal muscular atrophy (13, 39).

**Figure 3:**
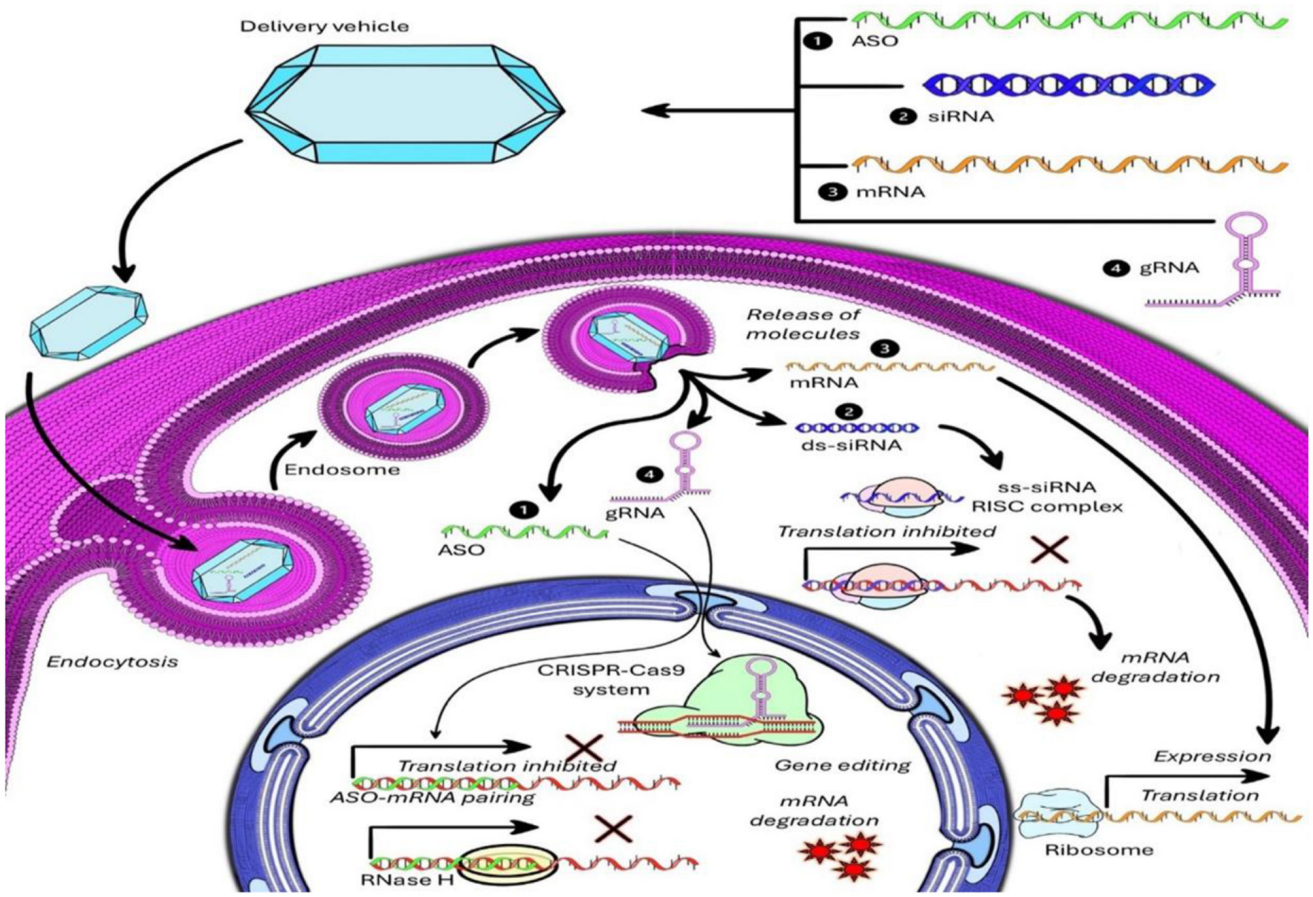
A schematic representation of intracellular delivery and gene modulation mechanisms of antisense oligonucleotides (ASO), small interfering RNA (siRNA), messenger RNA (mRNA), and guide RNA (gRNA) via a nanoparticle-based delivery system. The illustration demonstrates the journey and mechanisms of different nucleic acid-based therapeutics after their delivery into a target cell using a nanocarrier system. Delivery vehicle and cellular entry: a synthetic nanoparticle-based delivery vehicle encapsulates therapeutic molecules including ASO (green), siRNA (blue), mRNA (orange), and gRNA (pink). The delivery system undergoes endocytosis upon interacting with the cellular membrane, forming an endosome that facilitates internalization. Endosomal escape and cytoplasmic release: the nanoparticles escape the endosome, releasing their nucleic acid cargo into the cytoplasm, where the therapeutic actions are initiated. Mechanisms of action: (1) Antisense Oligonucleotide (ASO): single-stranded ASO binds to target mRNA via complementary base pairing. This pairing either inhibits translation directly by blocking ribosome binding or induces mRNA cleavage mediated by RNase H, leading to mRNA degradation and suppressed gene expression. (2) Small Interfering RNA (siRNA): double-stranded siRNA (ds-siRNA) is processed into single-stranded siRNA (ss-siRNA) and incorporated into the RNA-induced silencing complex (RISC). The RISC-siRNA complex binds to complementary mRNA, causing translation inhibition or mRNA degradation, effectively silencing gene expression. (3) Messenger RNA (mRNA): delivered mRNA is released in the cytoplasm and directly used by ribosomes for translation into functional proteins, thus promoting gene expression. (4) Guide RNA (gRNA) in CRISPR-Cas9 system: through base pairing, the gRNA guides the Cas9 endonuclease to a specific genomic locus. This enables gene editing through site-specific DNA cleavage, followed by either gene disruption or correction. The gRNA-Cas9 complex also contributes to mRNA degradation in some designs or inhibits transcription if used with catalytically inactive dCas9 fused to transcription repressors. The outcomes of these mechanisms result in either suppression (via ASO, siRNA, or CRISPR) or induction (via mRNA, or CRISPR) of gene expression, contributing to therapeutic effects tailored for diseases such as cancer, viral infections, and genetic disorders. Application of ASO is not limited to inhibition only (84).

Although ASOs use similar nucleotide chemistry, they are refined through various chemical modifications to improve nuclease resistance, reduce off-target effects, and enhance RNA binding. Common modifications include phosphorothioate linkages and 2'-O-methoxyethyl substitutions, which help improve half-life and biodistribution. Because of their mechanism, ASOs can reach intracellular RNA targets and are suitable for both systemic and central nervous system (CNS)-directed administration (Figure 3) (40). Several ASOs currently in clinical development are listed in Table 2.

**Table 2:** The current table explains different target genes and their therapeutic modalities.

**Sr. no**.	**Diseases**	**Target genes**	**Therapeutic goals**	**Therapeutic modality**
1.	Alzheimer's disease	APP/PSEN1/PSEN2	Helps to correct the mutation. Causative mutations for Early-Onset AD (EOAD); DRIVE A-beta overproduction.	Gene editing (CRISPR/Cas9)
		MAPT	Encodes tau protein; aggregation into neurofibrillary tangles (NFT's) correlates with cognitive decline. Reduces tau production.	ASO (gene silencing)
		APOE	Strongest genetic risk factor for Late-onset AD (LOAD). Converts APOE4 TO APOE3/2 or augment with protective APOE2.	Gene editing/gene augmentation (AAV)
		BDNF	Neurotrophic factor is essential for neuronal survival and plasticity; their levels are reduced in AD. Helps to restore BDNF levels.	Gene augmentation (AAV2)
2.	Parkinson's disease	SNCA	Encodes alpha-synuclein; aggregation is a key pathological hallmark. Used to reduce alpha-synuclein production.	ASO/AAV-RNAi
		LRRK2	Inhibits kinase activity. Familial and sporadic PD with hyperactive kinase function is caused by mutations.	ASO/small molecule
		GBA	To impair lysosomal function the most common genetic risk factor are mutations. Restores GCase enzyme activity.	Gene replacement (AAV9)
		AADC	Enzyme for synthesis of dopamine as its levels decline in PD. Enhances brain's ability to produce dopamine and levodopa.	Gene augmentation (AAV2)
3.	Huntington's disease	HTT	CAG repeats expansion in HTT gene creating a toxic mutant protein as mHTT. Used to lower mHTT levels or permanently inactivate the mutant HTT gene.	ASO (gene silencing), AAV-miRNA, small molecule splicing modulator, CRISPR/Cas9 or CRISPR/Cas13d
4.	Amyotrophic lateral sclerosis	SOD1	Causes toxic gain of function by mutations, which leads to protein aggregation and motor neural death. Used to reduce SOD1 protein.	ASO (gene silencing)
		C9orf72	Most common genetic cause of ALS is Hexanucleotide repeat expansion. Helps to degrade repeat-containing RNA transcripts.	ASO (gene silencing)
		FUS	Toxic gain of function occurs by mutation, often in aggressive young-onset ALS. Used to reduce FUS protein production.	ASO (gene silencing)
		TARDBP	Encodes TDP-43; protein mislocalization and aggregation is a hallmark of >97% of ALS cases.	AAV-Intrabody

## Stem-cell therapeutics

While genetic therapies address the upstream causes or contributors of neurodegeneration, cellular therapies aim to replace lost cells or modulate the disease environment via cell-derived factors. The brain's limited capacity for self-repair has made cell therapy an appealing strategy, especially for diseases characterized by the loss of a specific cell population (e.g., dopaminergic neurons in PD, motor neurons in ALS, oligodendrocytes in multiple sclerosis). Advances in stem cell technology, particularly the emergence of induced pluripotent stem cells (iPSCs), human embryonic stem cells (hESCs), mesenchymal stem cells (MSCs), and neural stem cells (NSCs), have accelerated therapeutic efforts across a spectrum of disorders.

### Parkinson's disease

Parkinson's disease is marked by the progressive degeneration of dopaminergic neurons in the substantia nigra pars compacta, has become the most advanced target of cell-based therapy. Traditional pharmacological interventions such as levodopa-carbidopa combinations and deep-brain stimulation offer symptomatic relief yet fail to halt neuronal degeneration and often lead to significant side effects like hallucinations, dyskinesia, and cognitive disturbances (41). Preclinical studies using hESCs or iPSCs differentiated into dopaminergic-producing neurons have demonstrated significant restoration of motor behaviors such as bradykinesia and tremor in PD models (42, 43). Clinically, a landmark phase I trial in Japan transplanted allogeneic iPSC-derived dopaminergic progenitors bilaterally into the putamen of seven PD patients (44). Two-year follow-up reported no serious adverse events (e.g., tumors or immune rejection), and PET imaging indicated new dopamine production in the striatum, with most patients showing functional improvement (45). A single-patient autologous iPSC transplant yielded comparable graft survival and motor benefits. Parallel trials, including one by BlueRock Therapeutics (NCT04802733) and another in Europe (STEM-PD, NCT05635409), are currently evaluating hESC-derived DA neuronal grafts. These studies represent the first clinical evidence that stem cell-derived midbrain DA neuron replacement is not only feasible and safe, but potentially disease-modifying (44, 46, 47).

### Alzheimer's disease

Alzheimer's disease in contrast, presents a more diffuse neurodegenerative pattern involving widespread neuronal loss in the cortex and hippocampus. The primary pathological features, including Aβ deposition and hyperphosphorylated tau accumulation (48), have proved resistant to conventional therapies like cholinesterase inhibitors and memantine. As a result, cell therapy efforts in AD have targeted both direct neuronal replacement and immune modulation. One promising avenue has focused on the transplantation of human umbilical cord blood-derived MSCs into the anterior hippocampus, which in small-scale studies was shown to be safe and offered cognitive benefit in AD patients (49–51). A Phase I trial in China (ChiCTR2000039011) is investigating hESC-derived NSCs injected into the hippocampus of AD patients (50). Additionally, a Phase 2a randomized trial using laromestrocel, an allogeneic bone marrow MSC product administered intravenously, showed that four monthly infusions led to 48% less whole-brain atrophy over 39 weeks and mild cognitive improvement vs. placebo (52). While these effects are modest, they suggest MSCs may act via anti-inflammatory and trophic pathways. Although true neuronal replacement for AD remains elusive, refining protocols for differentiating cortical neurons or interneurons could allow future cell-based circuit reconstruction.

### Amyotrophic lateral sclerosis

It involves the progressive degeneration of upper and lower motor neurons and presents a unique challenge for cell replacement due to the complexity of axonal architecture. Cell-based approaches have instead focused on glial or inter-neuronal support. Neuralstem Inc. used a fetal spinal cord-derived NSC line (NSI-566) injected directly into the spinal cord in Phase I–II trials (NCT01730716), finding the procedure safe with suggestions of slowed progression in some patients. A long-term follow-up hinted at increased survival compared to historical controls (53). In parallel, autologous MSCs engineered to secrete neurotrophic factors (NurOwn) were tested in a Phase 3 trial, which unfortunately did not meet its primary endpoint, although a *post-hoc* analysis suggested functional benefit in a less advanced subgroup (54). The aggressive progression of ALS may require early intervention or combinatorial strategies, but these studies underscore the potential of stem cell support for motor neuron preservation.

### Huntington's disease

It is defined by the selective loss of GABAergic medium spiny neurons in the striatum, making it an ideal candidate for neuronal replacement. Historical efforts using fetal striatal tissue achieved variable outcomes, but current research is focused on differentiating iPSCs into medium spiny neurons (55). In addition, a novel strategy involving MSC-derived exosomes loaded with therapeutic microRNAs has entered early-stage clinical evaluation, offering a paracrine approach to modulate mutant huntingtin expression and provide neuroprotection (56). The focal nature of HD neuropathology, combined with these evolving cellular tools, makes it a promising platform for future precision cell therapies (57).

## Monogenic vs. polygenic targets: tailoring therapeutics

Neurodegenerative diseases range from monogenic conditions—caused by mutations in a single gene—to complex polygenic disorders involving multiple risk alleles and environmental factors. Understanding this distinction is important for selecting appropriate therapeutic strategies (Table 2).

### Monogenic disorders

It includes Huntington's disease (HD), familial amyotrophic lateral sclerosis (ALS), familial Alzheimer's disease (AD), and several spinocerebellar ataxias. These diseases are linked to well-defined genetic mutations, such as the HTT expansion in HD or SOD1 and C9orf72 in ALS (58, 59). In these cases, gene-targeted interventions are feasible. Approaches include gene silencing (using ASOs, siRNA, or CRISPR) for toxic gain-of-function mutations, and gene supplementation in cases of loss-of-function (60). For example, the ASO tofersen targets mutant SOD1 in ALS, while AAV-based delivery of SMN1 is used to treat spinal muscular atrophy (SMA) (61, 62). In HD, lowering mutant HTT expression through ASOs has led to dose-dependent reduction in HTT protein in trials (63). In such disorders, the main challenges are delivery method and timing, rather than identifying the disease target (64).

Preventive strategies may also be possible in monogenic conditions. One trial is testing an ASO in presymptomatic SOD1 mutation carriers to evaluate whether disease onset can be delayed or prevented. Similar discussions are ongoing around early intervention in children carrying pathogenic APP or PSEN1 mutations (65). Regulatory development is often more straightforward for monogenic diseases, where biomarkers—such as mutant protein levels—can guide dosing and assess response with less variability.

### Polygenic and complex disorders

Polygenic disorders including sporadic AD, sporadic PD, and Lewy body dementia, do not involve a single causative gene. Although genetic risk factors exist—such as APOE in AD or LRRK2 in PD, these conditions involve overlapping pathophysiological processes. Therapeutic approaches focus on shared downstream mechanisms, such as protein aggregation, synaptic loss, and inflammation. For instance, tau-targeting ASOs are in trials for AD, and SNCA (α-synuclein) reduction strategies are under development for PD (66–68). Although these targets are not causative genes, they are strongly implicated in disease pathology.

Inflammation-related targets are also under consideration. For example, enhancing TREM2 activity may improve microglial clearance of amyloid plaques in AD. TREM2 variants increase AD risk, but microglial modulation may also benefit sporadic cases (69). In PD, delivery of PRKN or PINK1 via gene therapy is being investigated to support mitophagy, even in patients without mutations in these genes (70, 71).

Therapies for complex diseases may require combination approaches. In AD, a patient might benefit from concurrent treatments that reduce amyloid, suppress tau pathology, and modulate immune activation. This multi-target strategy reflects the multifactorial nature of disease progression. Trial design is also more difficult in polygenic diseases due to heterogeneity. Patient selection based on genotype, biomarkers, or polygenic risk scores may improve trial outcomes. For example, APOE4 carriers are being studied for AAV-based gene therapies that either suppress APOE4 or increase protective APOE2 expression (72, 73).

In conclusion, monogenic neurodegenerative diseases offer clear molecular targets and are more amenable to single-gene therapies. Many early gene therapy successes, such as in SMA and familial ALS, fall into this category. Polygenic diseases, on the other hand, require broader strategies aimed at modulating central pathways rather than correcting a single gene. As a result, partial efficacy from single agents may necessitate combined or sequential treatments to achieve significant clinical benefit.

## Discussion

Neurodegenerative diseases such as Alzheimer's disease (AD), Parkinson's disease (PD), Huntington's disease (HD), and amyotrophic lateral sclerosis (ALS) remain major therapeutic challenges due to their progressive nature, complex genetic and environmental underpinnings, and limited regenerative capacity of the central nervous system (CNS). Recent advances in gene therapy, RNA-based therapeutics, and cell-based interventions have begun to shift treatment paradigms—from symptomatic relief toward potential disease-modifying approaches.

In PD, degeneration of dopaminergic neurons in the substantia nigra leads to characteristic motor symptoms. Viral vector-based strategies have shown clinical potential, with AAV-mediated gene delivery targeting enzymes such as aromatic L-amino acid decarboxylase (AADC) and neurotrophic factors like GDNF or neurturin demonstrating promising results in early-phase trials (NCT01621581, NCT04167540, NCT00985517) (74, 75). Lentiviral approaches, including the ProSavin platform, have shown sustained expression of dopamine biosynthetic enzymes and clinical benefit in select cases. Complementing these approaches, stem cell-based therapies—particularly transplantation of iPSC- and hESC-derived dopaminergic progenitors—have demonstrated graft survival, dopaminergic function on PET imaging, and moderate motor improvements in early trials (42–45). These findings suggest that combining gene delivery with cell replacement may offer synergistic benefit by providing both enzymatic support and neuronal integration.

In AD, strategies have focused on modifying risk-related pathways. AAV-mediated delivery of the protective APOE2 allele is being explored in APOE4 carriers (76), while stem cell-derived MSC therapies such as laromestrocel have demonstrated reduced neuroinflammation and brain atrophy, albeit with modest cognitive improvements (52). These outcomes, though preliminary, highlight the feasibility of multimodal approaches targeting both genetic and inflammatory contributors.

RNA-based therapeutics have expanded rapidly, with ASO-based interventions targeting SOD1 in ALS (tofersen), tau in AD, and huntingtin in HD advancing to clinical testing (39, 40, 77). Success in spinal muscular atrophy has validated this class of drugs, though CNS delivery—typically via intrathecal administration—remains a key limitation. Nanoparticle-based methods are under exploration to improve biodistribution and reduce procedural burden. Despite progress, major translational hurdles remain. Crossing the blood-brain barrier effectively, maintaining long-term transgene expression, managing immunogenicity, and manufacturing scalable GMP-compliant vectors and cell products all require further optimization.

Overall, the past decade has marked substantial progress in diversifying the therapeutic arsenal for neurodegeneration. The convergence of viral gene therapy, ASO platforms, stem cell interventions, and genome editing tools has created opportunities for precision medicine. Going forward, combined and sequential treatment regimens—tailored to individual disease profiles—may be necessary to produce durable and functionally meaningful improvements. The following sections discuss emerging gene targets and explore frameworks for multimodal and adaptive neurotherapeutic strategies.

## Emerging targets in neurotherapeutics

Several gene targets and therapeutic innovations are now under investigation for their potential in neurodegenerative diseases:

TREM2 (Triggering receptor expressed on myeloid cells 2) is a microglial surface protein, where variants like R47H increase Alzheimer's disease (AD) risk by approximately threefold. TREM2 promotes microglial phagocytosis and survival in the presence of amyloids (69). In a mouse model, over-expression of TREM2 reduced amyloid plaque seeding and neuroinflammation (78). TREM2-activating antibodies are also in Phase I trials (e.g., Alector/AbbVie). If proven safe, gene-based methods to increase TREM2 function could support microglial response in early AD, especially in APOE4 carriers where microglial dysfunction is prominent (78).

Progranulin (GRN) mutations, which lower progranulin levels, are linked to frontotemporal dementia (FTD). Progranulin also has neurotrophic and immunomodulatory roles in other diseases, including AD, where higher levels correlate with slower degeneration. AAV-based GRN gene therapy is being evaluated in FTD-GRN mutation carriers. Prevail Therapeutics has initiated a Phase I trial delivering GRN via AAV9 into the cerebrospinal fluid. AVB-101 (AviadoBio) is another program that uses AAV to deliver GRN into the thalamus and is now entering clinical trials (79). If successful, these therapies may not only help mutation carriers but also support neuronal survival in broader neurodegenerative conditions.

GBA1 and lysosomal genes: Heterozygous mutations in GBA1, which encodes glucocerebrosidase (GCase), impair lysosomal function and increase the risk of Parkinson's disease (PD) and dementia with Lewy bodies. AAV-GBA therapy aiming to enhance GCase activity is in Phase I testing for PD (80). Other lysosomal regulators, such as TFEB—a key transcription factor controlling autophagy—are also under investigation as potential targets to improve protein aggregate clearance (81).

PINK1 and parkin (PRKN) are involved in mitophagy, the clearance of damaged mitochondria. Mutations in these genes cause familial PD, and mitochondrial impairment is also observed in sporadic PD. AAV-mediated overexpression of Parkin protected dopaminergic neurons from α-synuclein toxicity in preclinical models and improved motor performance in toxin-induced PD models (70, 71). A UCL-led program, supported by the Michael J. Fox Foundation, is testing an AAV carrying a modified “mini-Parkin” in rats, which showed improved motor recovery after toxin exposure (70). These tools may soon be evaluated in moderate-stage PD patients.

Alpha-Synuclein and tau remain central targets in future studies. Although not newly discovered, both SNCA (alpha-synuclein) and MAPT (tau) are key contributors to disease. ASOs and CRISPR-based methods to reduce SNCA expression are advancing toward trials in synucleinopathies (67, 68). Tau-targeting ASOs are already in human studies. If these interventions demonstrate acceptable safety and even partial efficacy, they could be paired with existing anti-amyloid therapies in AD.

## Challenges and outlook

Although gene, RNA-based, and stem cell therapies offer potential for treating neurodegenerative diseases, several challenges must be addressed before these approaches can be applied broadly in clinical settings. One major difficulty is efficient delivery, especially across the blood-brain barrier (BBB). While AAV9 and related capsids demonstrate some natural CNS tropism, many therapies still require direct delivery methods such as intrathecal or intracerebral injection due to limited distribution in the brain (52, 82). New strategies involving nanoparticle carriers and engineered AAV capsids are under development to improve CNS access, but most of them are still in early testing stages.

Another limitation is the complexity of large-scale manufacturing. Clinical-grade production of AAV vectors, ASOs, and gene editing materials requires strict quality control and carries high cost. Similarly, stem cell therapies, particularly those based on iPSCs or autologous cells—need customized protocols and GMP-compliant facilities, which increase logistical and regulatory burdens (Table 3).

**Table 3:** The current table explains advantages and disadvantages of different therapeutical aspects of neurodegenerative disorders.

**Sr. no**.	**Therapy types**	**Advantages**	**Disadvantages**
1.	Adeno associated virus	a. AAV viral vectors are easy to transduce and contain high efficiency for gene transfer. b. AAV viral vectors shows high efficiency for tissue-tropism for central nervous tissues.	a. AAVs mediated transduction provides transient expression. b. Pre-existing seroprevalence is available among individuals for AAV serotypes. c. AAVs impose off-target effects.
2.	Lentivirus	a. Lentivirus mediated transduction provides stable long term gene expression. b. Lentivirus show high efficiency against both neural originate and hematopoietic cells. c. These viruses capable of transducing large transgenes and (~9 kb) and immune evasion.	a. Lentiviruses possess severe virulence effects since they originated from HIV-origin backbone. b. Transducing cells using lentiviral mediated system do not allow wide range of cell types. c. Preparation of lentiviral for a specific trans-gene transduction is a tedious process and transducing efficiencies are very less.
3.	Stem cell therapeutics	a. Different stem cell types (Mesenchymal and embryonic) showed effective progress for major neurodegenerative disorders. b. Stem cells mediated therapeutical clinical trials provides long term benefits for several major neurodegenerative disorders.	a. Stem cells mediated therapies are complicate and require several regulatory procedures to be approved. b. Differentiating stem cells for neural origin might lead for complicated issues. c. The success rate for stem cell transplant is not efficient and often leads to major complications.
4.	Short oligonucleotides	a. Anti-sense oligonucleotides provide long tern effects in major neurodegenerative disorders. b. The delivery of anti-sense oligonucleotide is comparatively efficient and do not require large preparations. c. The synthesis of short-oligos against over-expressive or mutant genes is highly efficient and less cumbersome process.	a. Oligos might lead for non-specific gene silencing causing adverse effects. a. Exo and endo nucleases present within cytoplasm might lead for non-specific degradation of therapeutic oligos. c. The endosomal mediated degradation of Oligos is frequent with this method of treatment.

Patient selection also presents a challenge. In monogenic diseases like SOD1-related ALS or Huntington's disease, a known mutation can be directly targeted. However, polygenic and sporadic conditions such as Alzheimer's and Parkinson's involve multiple genes and environmental factors. Identifying the right patient groups based on genotype, disease stage, or biomarkers is important for improving trial outcomes, but this process remains complex (52, 76).

Finally, long-term data on safety and efficacy are limited. Although short-term results show safety for several gene and cell therapies, there are still concerns regarding immune responses, off-target effects, and sustained expression (2, 83). For example, gene editing methods may introduce unintended mutations or trigger chronic inflammation. Continued clinical observation and improved preclinical models will be necessary to optimize delivery, monitor immune outcomes, and evaluate therapeutic stability.

## Conclusion

The therapeutic landscape for neurodegenerative disorders is undergoing a profound transformation, driven by advances in gene therapy, RNA-based therapeutics, and cell-based interventions. Traditional pharmacological approaches have largely failed to modify the course of diseases like Alzheimer's, Parkinson's, and ALS. In contrast, emerging modalities—such as AAV- and lentivirus-mediated gene delivery, antisense oligonucleotides, and stem cell-based strategies—offer the potential for more precise, targeted, and durable treatments.

This review highlights how these technologies are being applied across both monogenic and polygenic neurodegenerative diseases, with promising results in early-phase trials. We also emphasize the growing relevance of genome editing tools, BBB-permeable delivery systems, and combinatorial approaches that integrate gene modulation with cellular regeneration. While several of these therapies remain in experimental stages, their ability to directly address disease mechanisms marks a paradigm shift in the treatment of neurodegeneration.

Continued innovation in delivery platforms, target validation, and clinical trial design will be essential for translating these emerging therapies into safe, effective, and accessible treatments. As the field matures, convergence of gene- and cell-based modalities holds the greatest promise for redefining long-term disease outcomes in neurodegenerative medicine.

## References

[1] Burnham SC Laws SM Budgeon CA Doré V Porter T Bourgeat P . Impact of APOE-ε4 carriage on the onset and rates of neocortical Aβ-amyloid deposition. Neurobiol Aging. (2020) 95:46. doi: 10.1016/j.neurobiolaging.2020.06.001 32750666 PMC7609543

[2] Abeliovich A Hefti F Sevigny J. Gene therapy for Parkinson's disease associated with GBA1 mutations. J Parkinsons Dis. (2021) 11(Suppl. 2):S183–8. doi: 10.3233/JPD-212739 34151863 PMC8543272

[3] Prince M Bryce R Albanese E Wimo A Ribeiro W Ferri CP. The global prevalence of dementia: a systematic review and metaanalysis. Alzheimers Dement. (2013) 9:63–75.e2. doi: 10.1016/j.jalz.2012.11.007 23305823

[4] Sagar R Dandona R Gururaj G Dhaliwal RS Singh A Ferrari A . The burden of mental disorders across the states of India: the Global Burden of Disease Study 1990–2017. Lancet Psychiatry. (2020) 7:148–61. doi: 10.1016/S2215-0366(19)30475-4 31879245 PMC7029418

[5] Brommel CM Cooney AL Sinn PL. Adeno-associated virus-based gene therapy for lifelong correction of genetic disease. Hum Gene Ther. (2020) 31:985–95. doi: 10.1089/hum.2020.138 32718227 PMC7495917

[6] Chen KS Koubek EJ Sakowski SA Feldman EL. Stem cell therapeutics and gene therapy for neurologic disorders. Neurotherapeutics. (2024) 21:e00427. doi: 10.1016/j.neurot.2024.e00427 39096590 PMC11345629

[7] Issa SS Shaimardanova AA Solovyeva VV Rizvanov AA. Various AAV serotypes and their applications in gene therapy: an overview. Cells. (2023) 12:785. doi: 10.3390/cells12050785 36899921 PMC10000783

[8] Cartier N Hacein-Bey-Abina S Bartholomae CC Veres G Schmidt M Kutschera I . Hematopoietic stem cell gene therapy with a lentiviral vector in X-linked adrenoleukodystrophy. Science. (2009) 326:818–23. doi: 10.1126/science.1171242 19892975

[9] Palfi S Gurruchaga JM Ralph GS Lepetit H Lavisse S Buttery PC . Long-term safety and tolerability of ProSavin, a lentiviral vector-based gene therapy for Parkinson's disease: a dose escalation, open-label, phase 1/2 trial. Lancet. (2014) 383:1138–46. doi: 10.1016/S0140-6736(13)61939-X 24412048

[10] Giannelli SG Luoni M Iannielli A Middeldorp J Philippens I Bido S . New AAV9 engineered variants with enhanced neurotropism and reduced liver off-targeting in mice and marmosets. iScience. (2024) 27:109777. doi: 10.1016/j.isci.2024.109777 38711458 PMC11070337

[11] Wang D Li S Gessler DJ Xie J Zhong L Li J . A rationally engineered capsid variant of AAV9 for systemic CNS-directed and peripheral tissue-detargeted gene delivery in neonates. Mol Ther Methods Clin Dev. (2018) 9:234. doi: 10.1016/j.omtm.2018.03.004 29766031 PMC5948233

[12] Ou K Jia Q Li D Li S Li XJ Yin P. Application of antisense oligonucleotide drugs in amyotrophic lateral sclerosis and Huntington's disease. Transl Neurodegener. (2025) 14:4. doi: 10.1186/s40035-025-00466-9 39838446 PMC11748355

[13] Mansour HM El-Khatib AS. Oligonucleotide-based therapeutics for neurodegenerative disorders: focus on antisense oligonucleotides. Eur J Pharmacol. (2025) 998:177529. doi: 10.1016/j.ejphar.2025.177529 40118328

[14] Khandia R Gurjar P. Priyanka, Romashchenko V, Al-Hussain SA, Zaki MEA. Recent advances in stem cell therapy: efficacy, ethics, safety concerns, and future directions focusing on neurodegenerative disorders—a review. Int J Surg. (2024) 110:6367–81. doi: 10.1097/JS9.0000000000001609 39705668 PMC11486982

[15] Temple S. Advancing cell therapy for neurodegenerative diseases. Cell Stem Cell. (2023) 30:512–29. doi: 10.1016/j.stem.2023.03.017 37084729 PMC10201979

[16] Wang JH Gessler DJ Zhan W Gallagher TL Gao G. Adeno-associated virus as a delivery vector for gene therapy of human diseases. Signal Transduct Target Ther. (2024) 9:78. doi: 10.1038/s41392-024-01780-w 38565561 PMC10987683

[17] Sen D. Improving clinical efficacy of adeno associated vectors by rational capsid bioengineering. J Biomed Sci. (2014) 21:103. doi: 10.1186/s12929-014-0103-1 25425174 PMC4251935

[18] Castle MJ Turunen HT Vandenberghe LH Wolfe JH. Controlling AAV tropism in the nervous system with natural and engineered capsids. Methods Mol Biol. (2016) 1382:133–49. doi: 10.1007/978-1-4939-3271-9_10 26611584 PMC4993104

[19] Liu S Li J Peraramelli S Luo N Chen A Dai M . Systematic comparison of rAAV vectors manufactured using large-scale suspension cultures of Sf9 and HEK293 cells. Mol Ther. (2024) 32:74–83. doi: 10.1016/j.ymthe.2023.11.022 37990495 PMC10787191

[20] Flotte TR Trapnell BC Humphries M Carey B Calcedo R Rouhani F . Phase 2 clinical trial of a recombinant adeno-associated viral vector expressing α 1-antitrypsin: interim results. Hum Gene Ther. (2011) 22:1239–47. doi: 10.1089/hum.2011.053 21609134 PMC3205788

[21] Manfredsson FP Rising AC Mandel RJ. AAV9: a potential blood-brain barrier buster. Mol Ther. (2009) 17:403. doi: 10.1038/mt.2009.15 19247366 PMC2835088

[22] Stanimirovic DB Sandhu JK Costain WJ. Emerging technologies for delivery of biotherapeutics and gene therapy across the blood–brain barrier. BioDrugs. (2018) 32:547–59. doi: 10.1007/s40259-018-0309-y 30306341 PMC6290705

[23] Pietersz KL Plessis FD Pouw SM Liefhebber JM van Deventer SJ Martens GJM . PhP.B enhanced adeno-associated virus mediated-expression following systemic delivery or direct brain administration. Front Bioeng Biotechnol. (2021) 9:679483. doi: 10.3389/fbioe.2021.679483 34414171 PMC8370029

[24] Chauhan M Daugherty AL Khadir FE Duzenli OF Hoffman A Tinklenberg JA . AAV-DJ is superior to AAV9 for targeting brain and spinal cord, and de-targeting liver across multiple delivery routes in mice. J Transl Med. (2024) 22:824. doi: 10.1186/s12967-024-05599-5 39237935 PMC11375878

[25] Ye D Chukwu C Yang Y Hu Z Chen H. Adeno-associated virus vector delivery to the brain: technology advancements and clinical applications. Adv Drug Deliv Rev. (2024) 211:115363. doi: 10.1016/j.addr.2024.115363 38906479 PMC11892011

[26] Selles MC Fortuna JTS Cercato MC Santos LE Domett L Bitencourt ALB . AAV-mediated neuronal expression of an scFv antibody selective for Aβ oligomers protects synapses and rescues memory in Alzheimer models. Mol Ther. (2023) 31:409–19. doi: 10.1016/j.ymthe.2022.11.002 36369741 PMC9931599

[27] Sondhi D Kaminsky SM Hackett NR Pagovich OE Rosenberg JB De BP . Slowing late infantile Batten disease by direct brain parenchymal administration of a rh.10 adeno-associated virus expressing CLN2. Sci Transl Med. (2020) 12:eabb5413. doi: 10.1126/scitranslmed.abb5413 33268510 PMC8056991

[28] White KA Nelvagal HR Poole TA Lu B Johnson TB Davis S . Intracranial delivery of AAV9 gene therapy partially prevents retinal degeneration and visual deficits in CLN6-Batten disease mice. Mol Ther Methods Clin Dev. (2021) 20:497–507. doi: 10.1016/j.omtm.2020.12.014 33665223 PMC7887332

[29] Elder JB. AADC gene therapy for Parkinson's disease. In:Tuszynski MH, editor. Translational Neuroscience. Berlin: Springer Nature (2025). p. 81–99. doi: 10.1007/978-3-031-89307-0_6

[30] Pearson TS Gupta N San Sebastian W Imamura-Ching J Viehoever A Grijalvo-Perez A . Gene therapy for aromatic L-amino acid decarboxylase deficiency by MR-guided direct delivery of AAV2-AADC to midbrain dopaminergic neurons. Nat Commun. (2021) 12:4251. doi: 10.1038/s41467-021-24524-8 34253733 PMC8275582

[31] Reilly A Yaworski R Beauvais A Schneider BL Kothary R. Long term peripheral AAV9-SMN gene therapy promotes survival in a mouse model of spinal muscular atrophy. Hum Mol Genet. (2024) 33:510–9. doi: 10.1093/hmg/ddad202 38073249 PMC10908349

[32] Srinivasakumar N. HIV-1 vector systems. Somat Cell Mol Genet. (2001) 26:51–81. doi: 10.1023/A:1021074613196 12465462

[33] Amirache F Lévy C Costa C Mangeot PE Torbett BE Wang CX . Mystery solved: VSV-G-LVs do not allow efficient gene transfer into unstimulated T cells, B cells, and HSCs because they lack the LDL receptor. Blood. (2014) 123:1422–4. doi: 10.1182/blood-2013-11-540641 24578496

[34] Finkelshtein D Werman A Novick D Barak S Rubinstein M. LDL receptor and its family members serve as the cellular receptors for vesicular stomatitis virus. Proc Natl Acad Sci USA. (2013) 110:7306–11. doi: 10.1073/pnas.1214441110 23589850 PMC3645523

[35] Vannucci L Lai M Chiuppesi F Ceccherini-Nelli L Pistello M. Viral vectors: a look back and ahead on gene transfer technology. New Microbiol. (2021) 36:1–22. Available online at: https://pubmed.ncbi.nlm.nih.gov/23435812/ 23435812

[36] Blömer U Kafri T Randolph-Moore L Verma IM Gage FH. Bcl-xL protects adult septal cholinergic neurons from axotomized cell death. Proc Natl Acad Sci USA. (1998) 95:2603–8. doi: 10.1073/pnas.95.5.2603 9482933 PMC19429

[37] Palfi S Gurruchaga JM Lepetit H Howard K Ralph GS Mason S . Long-term follow-up of a phase I/II study of ProSavin, a lentiviral vector gene therapy for Parkinson's disease. Hum Gene Ther Clin Dev. (2018) 29:148. doi: 10.1089/humc.2018.081 30156440 PMC6157351

[38] Miao Y Fu C Yu Z Yu L Tang Y Wei M. Current status and trends in small nucleic acid drug development: leading the future. Acta Pharm Sin B. (2024) 14:3802–17. doi: 10.1016/j.apsb.2024.05.008 39309508 PMC11413693

[39] Leavitt BR Tabrizi SJ. Antisense oligonucleotides for neurodegeneration. Science. (2020) 367:1428–9. doi: 10.1126/science.aba4624 32217715

[40] Testa CM. Antisense oligonucleotide therapeutics for neurodegenerative disorders. Curr Geriatr Rep. (2022) 11:19–32. doi: 10.1007/s13670-020-00341-7

[41] Muranova A Shanina E. Levodopa/Carbidopa/Entacapone Combination Therapy. Treasure Island, FL: StatPearls (2023). 38261678

[42] Cha Y Park TY Leblanc P Kim KS. Current status and future perspectives on stem cell-based therapies for Parkinson's disease. J Mov Disord. (2023) 16:22. doi: 10.14802/jmd.22141 36628428 PMC9978267

[43] Cardoso T Adler AF Mattsson B Hoban DB Nolbrant S Wahlestedt JN . Target-specific forebrain projections and appropriate synaptic inputs of hESC-derived dopamine neurons grafted to the midbrain of parkinsonian rats. J Comp Neurol. (2018) 526:2133–46. doi: 10.1002/cne.24500 30007046 PMC6175216

[44] Nakamura R Nonaka R Oyama G Jo T Kamo H Nuermaimaiti M . A defined method for differentiating human iPSCs into midbrain dopaminergic progenitors that safely restore motor deficits in Parkinson's disease. Front Neurosci. (2023) 17:1202027. doi: 10.3389/fnins.2023.1202027 37502682 PMC10368972

[45] Chang JW Na HK Chang KW Park CW Kim DH Park S . Phase 1/2a clinical trial of hESC-derived dopamine progenitors in Parkinson's disease. Cell. (2025) 188:S0092-8674(25)01041-4. doi: 10.1016/j.cell.2025.09.010 41086804

[46] O'Leary K. Progress with stem cell therapy in Parkinson's disease. Nat Med. (2025). doi: 10.1038/d41591-025-00029-5. [Epub ahead of print]. 40307484

[47] Wang YK Zhu WW Wu MH Wu YH Liu ZX Liang LM . Human clinical-grade parthenogenetic ESC-derived dopaminergic neurons recover locomotive defects of nonhuman primate models of Parkinson's disease. Stem Cell Reports. (2018) 11:171–82. doi: 10.1016/j.stemcr.2018.05.010 29910127 PMC6067059

[48] Han F Bi J Qiao L Arancio O. Stem cell therapy for Alzheimer's disease. Adv Exp Med Biol. (2020) 1266:39–55. doi: 10.1007/978-981-15-4370-8_4 33105494

[49] Zhidu S Ying T Rui J Chao Z. Translational potential of mesenchymal stem cells in regenerative therapies for human diseases: challenges and opportunities. Stem Cell Res Ther. (2024) 15:266. doi: 10.1186/s13287-024-03885-z 39183341 PMC11346273

[50] Kang JM Yeon BK Cho SJ Suh YH. Stem cell therapy for Alzheimer's disease: a review of recent clinical trials. J Alzheimers Dis. (2016) 54:879–89. doi: 10.3233/JAD-160406 27567851

[51] Kim HJ Seo SW Chang JW Lee JI Kim CH Chin J . Stereotactic brain injection of human umbilical cord blood mesenchymal stem cells in patients with Alzheimer's disease dementia: a phase 1 clinical trial. Alzheimers Dement. (2015) 1:95–102. doi: 10.1016/j.trci.2015.06.007 29854930 PMC5975048

[52] Rash BG Ramdas KN Agafonova N Naioti E McClain-Moss L Zainul Z . Allogeneic mesenchymal stem cell therapy with laromestrocel in mild Alzheimer's disease: a randomized controlled phase 2a trial. Nat Med. (2025) 31:1257–66. doi: 10.1038/s41591-025-03559-0 40065171 PMC12003194

[53] Goutman SA Brown MB Glass JD Boulis NM Johe K Hazel T . Long-term phase 1/2 intraspinal stem cell transplantation outcomes in ALS. Ann Clin Transl Neurol. (2018) 5:730–40. doi: 10.1002/acn3.567 29928656 PMC5989736

[54] Berry JD Cudkowicz ME Windebank AJ Staff NP Owegi M Nicholson K . NurOwn, phase 2, randomized, clinical trial in patients with ALS: safety, clinical, and biomarker results. Neurology. (2019) 93:E2294–305. doi: 10.1212/WNL.0000000000008620 31740545 PMC6937497

[55] Ehrlich ME. Huntington's disease and the striatal medium spiny neuron: cell-autonomous and non-cell-autonomous mechanisms of disease. Neurotherapeutics. (2012) 9:270. doi: 10.1007/s13311-012-0112-2 22441874 PMC3337013

[56] Ananbeh H Vodicka P Kupcova Skalnikova H. Emerging roles of exosomes in Huntington's disease. Int J Mol Sci. (2021) 22:4085. doi: 10.3390/ijms22084085 33920936 PMC8071291

[57] Liang XS Sun ZW Thomas AM Li S. Mesenchymal stem cell therapy for Huntington disease: a meta-analysis. Stem Cells Int. (2023) 2023:1109967. doi: 10.1155/2023/1109967 37168444 PMC10164866

[58] Colombo E Poletti B Maranzano A Peverelli S Solca F Colombrita C . Motor, cognitive and behavioural profiles of C9orf72 expansion-related amyotrophic lateral sclerosis. J Neurol. (2022) 270:898–908. doi: 10.1007/s00415-022-11433-z 36308529 PMC9886586

[59] Jih KY Lai KL Lin KP Liao YC Lee YC. Reduced-penetrance Huntington's disease-causing alleles with 39 CAG trinucleotide repeats could be a genetic factor of amyotrophic lateral sclerosis. J Chin Med Assoc. (2023) 86:47–51. doi: 10.1097/JCMA.0000000000000837 36599142 PMC12755495

[60] Cantara S Simoncelli G Ricci C. Antisense oligonucleotides (ASOs) in motor neuron diseases: a road to cure in light and shade. Int J Mol Sci. (2024) 25:4809. doi: 10.3390/ijms25094809 38732027 PMC11083842

[61] Van Daele SH Masrori P Van Damme P Van Den Bosch L. The sense of antisense therapies in ALS. Trends Mol Med. (2024) 30:252–62. doi: 10.1016/j.molmed.2023.12.003 38216448

[62] Ludolph A Wiesenfarth M. Tofersen and other antisense oligonucleotides in ALS. Ther Adv Neurol Disord. (2025) 18:17562864251313915. doi: 10.1177/17562864251313915 39845577 PMC11752197

[63] Caron NS Aly AE Findlay Black H Martin DDO Schmidt ME Ko S . Systemic delivery of mutant huntingtin lowering antisense oligonucleotides to the brain using apolipoprotein A-I nanodisks for Huntington disease. J Control Release. (2024) 367:27–44. doi: 10.1016/j.jconrel.2024.01.011 38215984

[64] Rook ME Southwell AL. Antisense oligonucleotide therapy: from design to the Huntington disease clinic. Biodrugs. (2022) 36:105. doi: 10.1007/s40259-022-00519-9 35254632 PMC8899000

[65] Sumner CJ Miller TM. The expanding application of antisense oligonucleotides to neurodegenerative diseases. J Clin Invest. (2024) 134:e186116. doi: 10.1172/JCI186116 39352381 PMC11444189

[66] Mummery CJ Börjesson-Hanson A Blackburn DJ Vijverberg EGB De Deyn PP Ducharme S . Tau-targeting antisense oligonucleotide MAPTRx in mild Alzheimer's disease: a phase 1b, randomized, placebo-controlled trial. Nat Med. (2023) 29:1437–47. doi: 10.1038/s41591-023-02326-3 37095250 PMC10287562

[67] Cole TA Zhao H Collier TJ Sandoval I Sortwell CE Steece-Collier K . α-Synuclein antisense oligonucleotides as a disease-modifying therapy for Parkinson's disease. JCI Insight. (2021) 6:e135633. doi: 10.1172/jci.insight.135633 33682798 PMC8021121

[68] Sun Z Kantor B Chiba-Falek O. Neuronal-type-specific epigenome editing to decrease SNCA expression: implications for precision medicine in synucleinopathies. Mol Ther Nucleic Acids. (2024) 35:102084. doi: 10.1016/j.omtn.2023.102084 38130373 PMC10732167

[69] Li Y Xu H Wang H Yang K Luan J Wang S. TREM2: potential therapeutic targeting of microglia for Alzheimer's disease. Biomed Pharmacother. (2023) 165:115218. doi: 10.1016/j.biopha.2023.115218 37517293

[70] Lee S Kang M Lee S Yoon S Cho Y Min D . AAV-aMTD-Parkin, a therapeutic gene delivery cargo, enhances motor and cognitive functions in Parkinson's and Alzheimer's diseases. Pharmacol Res. (2024) 208:107326. doi: 10.1016/j.phrs.2024.107326 39069196

[71] Lo Bianco C Schneider BL Bauer M Sajadi A Brice A Iwatsubo T . Lentiviral vector delivery of parkin prevents dopaminergic degeneration in an α-synuclein rat model of Parkinson's disease. Proc Natl Acad Sci USA. (2004) 101:17510. doi: 10.1073/pnas.0405313101 15576511 PMC536019

[72] Chen Y Jin H Chen J Li J Găman MA Zou Z. The multifaceted roles of apolipoprotein E4 in Alzheimer's disease pathology and potential therapeutic strategies. Cell Death Discovery. (2025) 11:312. doi: 10.1038/s41420-025-02600-y 40628716 PMC12238274

[73] Li X Li Z Chen H Guo H Ge Y Dong F . Unraveling APOE4: the dual role in CNS and peripheral inflammation in Alzheimer's disease. Int Immunopharmacol. (2025) 163:115199. doi: 10.1016/j.intimp.2025.115199 40652586

[74] Bartus RT Baumann TL Siffert J Herzog CD Alterman R Boulis N . Safety/feasibility of targeting the substantia nigra with AAV2-neurturin in Parkinson patients. Neurology. (2013) 80:1698–701. doi: 10.1212/WNL.0b013e3182904faa 23576625 PMC3716474

[75] Manfredsson FP Polinski NK Subramanian T Boulis N Wakeman DR Mandel RJ. The future of GDNF in Parkinson's disease. Front Aging Neurosci. (2020) 12:593572. doi: 10.3389/fnagi.2020.593572 33364933 PMC7750181

[76] Sharma S Joshi V Kumar V. Implications of AAV serotypes in neurological disorders: current clinical applications and challenges. Clin Transl Neurosci. (2025) 9:32. doi: 10.3390/ctn9030032

[77] Scoles DR Minikel EV Pulst SM. Antisense oligonucleotides: a primer. Neurol Genet. (2019) 5:e323. doi: 10.1212/NXG.0000000000000323 31119194 PMC6501637

[78] Zhao N Qiao W Li F Ren Y Zheng J Martens YA . Elevating microglia TREM2 reduces amyloid seeding and suppresses disease-associated microglia. J Exp Med. (2022) 219:e20212479. doi: 10.1084/jem.20212479 36107206 PMC9481739

[79] AviadoBio: revolutionary gene therapies for neurodegenerative disorders. Available online at: https://www.nature.com/articles/d43747-023-00115-y (Accessed November 2, 2025).

[80] Ressler R McDowell K Wheeler B Thomas S Grigoryeva L Armendariz A . Systemic AAV gene therapy using a CNS-targeted engineered capsid significantly increases GCase activity to support the potential treatment of PD-GBA (S37.007). Neurology. (2025) 104(7 Suppl. 1):5149. doi: 10.1212/WNL.0000000000212169

[81] Franco-Juárez B Coronel-Cruz C Hernández-Ochoa B Gómez-Manzo S Cárdenas-Rodríguez N Arreguin-Espinosa R . TFEB; Beyond its role as an autophagy and lysosomes regulator. Cells. (2022) 11:3153. doi: 10.3390/cells11193153 36231114 PMC9562866

[82] Ittner LM Klugmann M Ke YD. Adeno-associated virus-based Alzheimer's disease mouse models and potential new therapeutic avenues. Br J Pharmacol. (2019) 176:3649. doi: 10.1111/bph.14637 30817847 PMC6715621

[83] Chen W Hu Y Ju D. Gene therapy for neurodegenerative disorders: advances, insights and prospects. Acta Pharm Sin B. (2020) 10:1347–59. doi: 10.1016/j.apsb.2020.01.015 32963936 PMC7488363

[84] Gupta A Andresen JL Manan RS Langer R. Nucleic acid delivery for therapeutic applications. Adv Drug Deliv Rev. (2021) 178:113834. doi: 10.1016/j.addr.2021.113834 34492233

